# Behavioural Correlate of Choice Confidence in a Discrete Trial Paradigm

**DOI:** 10.1371/journal.pone.0026863

**Published:** 2011-10-27

**Authors:** Doron Lavan, James S. McDonald, R. Frederick Westbrook, Ehsan Arabzadeh

**Affiliations:** School of Psychology, The University of New South Wales, Sydney, New South Wales, Australia; University of Sydney, Australia

## Abstract

How animals make choices in a changing and often uncertain environment is a central theme in the behavioural sciences. There is a substantial literature on how animals make choices in various experimental paradigms but less is known about the way they assess a choice after it has been made in terms of the expected outcome. Here, we used a discrete trial paradigm to characterise how the reward history shaped the behaviour on a trial by trial basis. Rats initiated each trial which consisted of a choice between two drinking spouts that differed in their probability of delivering a sucrose solution. Critically, sucrose was delivered after a delay from the first lick at the spouts – this allowed us to characterise the behavioural profile during the window between the time of choice and its outcome. Rats' behaviour converged to optimum choice, both during the acquisition phase and after the reversal of contingencies. We monitored the post-choice behaviour at a temporal precision of 1 millisecond; lick-response profiles revealed that rats spent more time at the spout with the higher reward probability and exhibited a sparser lick pattern. This was the case when we exclusively examined the unrewarded trials, where the outcome was identical. The differential licking profiles preceded the differential choice ratios and could thus predict the changes in choice behaviour.

## Introduction

The way animals make decisions that have uncertain variable outcomes can be investigated through various approaches. Optimal foraging theory presents the question from the Darwinian perspective – animals ought to optimise food yield, or maximum energy gained per unit of time [1]. To achieve this consistently, they must monitor the distribution of resources in their environment, update this information as these resources change, and adjust their selection of environmental locations appropriately as well as the time allocated to each chosen location [2]. There is behavioural evidence for optimal foraging [3], [4], and of its potential neuronal substrates [5]. Beyond foraging theory, other experimental paradigms have manipulated reinforcement rates and probabilities while studying the rules that govern choices [6], [7], [8], [9]. Example descriptions of choice between options with variable outcomes include Herrnstein's matching law [10], the local matching law [8], and the actor-critic algorithm (see for instance, [11]).

In spite of the substantial literature on how animals make choices in various experimental paradigms, in terms of patch selection or in terms of reinforcement rates, little is known about the way animals assess a choice after it has been made in terms of the possible outcome and its timing. Here we quantified aspects of the behavioural response to variability both before a choice is made and after. In particular, we identified an explicit representation of the animal's confidence in the outcome, as manifested in the post-choice behaviour. We used a discrete trial choice paradigm where rats chose between two options on every trial. Critically, the outcome of their choice was revealed after a variable time window. This allowed us to characterise the behaviour of the rats during that time window and search for behavioural correlates of reward expectancy.

## Results

**Figure 1:**
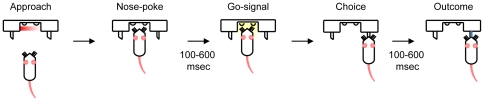
The behavioural sequence. Rats approach the aperture and break the sensor beam (nose-poke) which triggers the rest of the sequence. There is an initial delay of between 100 and 600 ms, during which the rat must maintain the nose-poke. Then a “go” signal is given, by the lighting up of two LEDs. The rat is then free to make a choice between the two spouts. The rats' spout choice is detected as soon as they lick a spout. The reward is then delivered with a probability determined by the reward rate (70% or 30%). The delivery of reward is delayed by a variable time (uniformly selected from 100–600 msec) and is independent from trial to trial.

Rats initiated a trial and received a go-signal which was then followed by a free choice between two options – a right or left drinking spout each of which could deliver a reward (a sucrose solution). Figure 1 illustrates the sequence of behavioural events. The initial reward contingencies were fixed across trials and sessions with a probability of 0.7 or 0.3 (the side of high probability reward counterbalanced across 4 rats). The rewards were delivered after a delay from the first lick at the spouts. The delay was the same for the high and low probability sides and was selected from a uniform distribution between 100–600 msec. All behavioural actions (i.e. nose-poke and contacts at the spout) and events (i.e. the go-signal and running of the pumps) were recorded at msec precision, across rewarded and unrewarded trials.

**Figure 2:**
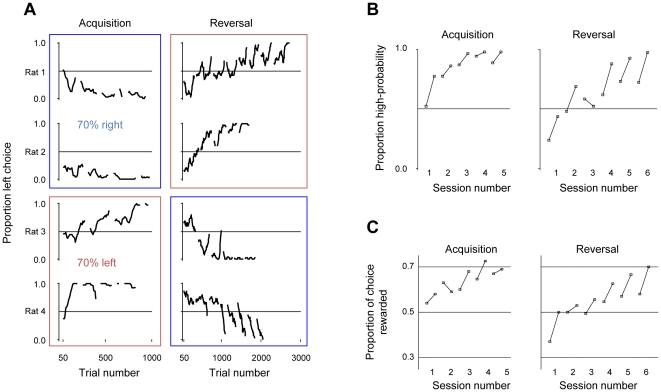
Rats' choice over all trials and sessions. A. Each row represents one rat. The left column represents the first 5 sessions (acquisition) while the right column represents reversal (6–8 sessions across rats). Each data point represents the average of the last 50 trials, spaced by 10 trial intervals. Breaks in the graphs indicate the start of a new session. Rats 1 and 2 (top rectangle) started with 70% reward left and 30% reward right. Rats 3 and 4 (bottom rectangle) had the inverse rewards. Only the first 1000 trials are plotted for acquisition. B. The proportion for which the high reward side was chosen, as a function of session number, averaged across all rats. Each pair of connected points represents the data for the first 50 and last 50 trials in a session. Left column represents acquisition sessions, right column the reversal sessions. C. The proportion of trials for which the rats were rewarded for their choice, as a function of session number, averaged across all rats. Each pair of connected points represents the data for the first 50 and last 50 trials in a session. Left column represents acquisition session, right column the reversal sessions.

Figure 2A shows the spout choice proportions separately for each of the four rats. Rats started with no systematic preference for either side, but eventually converged on nearly exclusive choice of the high probability option (the right spout for rats 1 and 2, and the left spout for rats 3 and 4). The convergence occurred across 5 sessions (a total of 800–1400 trials for different rats). After performing at a stable level of choice (>95% of choice to the high reward side across 100 trials), the contingencies were reversed. As illustrated in the second column of Figure 2A, rats switched their choices back to the optimum behaviour (>95% choice of the new high probability reward) after 6 to 8 sessions (2800 to 3700 trials for different rats). The convergence on exclusive choice of the high probability reward, known as maximising, took longer, in all four rats, for reversal compared to acquisition (on average 1076 trials longer). At the outset of every session, the rats showed a tendency to sample the low probability sides, which declined over the course of a session (beginning of sessions are indicated with breaks in the graphs of Figure 2A). This is most evident in the comparison of choice ratios early versus late in a trial as illustrated in Figure 2B. To characterise the efficiency of the behavioural choice, we estimated reward gained across trials relative to the performance of an ideal observer who was given the probabilities at the onset of every trial. The rats' choices of spout determined the quantity of reward they earned. By choosing only the high reward spout, maximising their behaviour, the rats would expect to earn reward for, on average, 70% of their choices (top horizontal line in Figure 2C). Proportion of reward choices, at the beginning and end of each session, is illustrated in Figure 2C. The rats started from an average 50% reward gain (i.e. reflecting approximately equal choice of the two sides) and over the course of three sessions arrived at a reward gain close to the optimum. At the outset of the reversal the rats nearly exclusively sampled the low reward side which resulted in a reward gain close to 30%. Subsequently they switched their choice and reached near optimum performance after 6 days.

**Figure 3:**
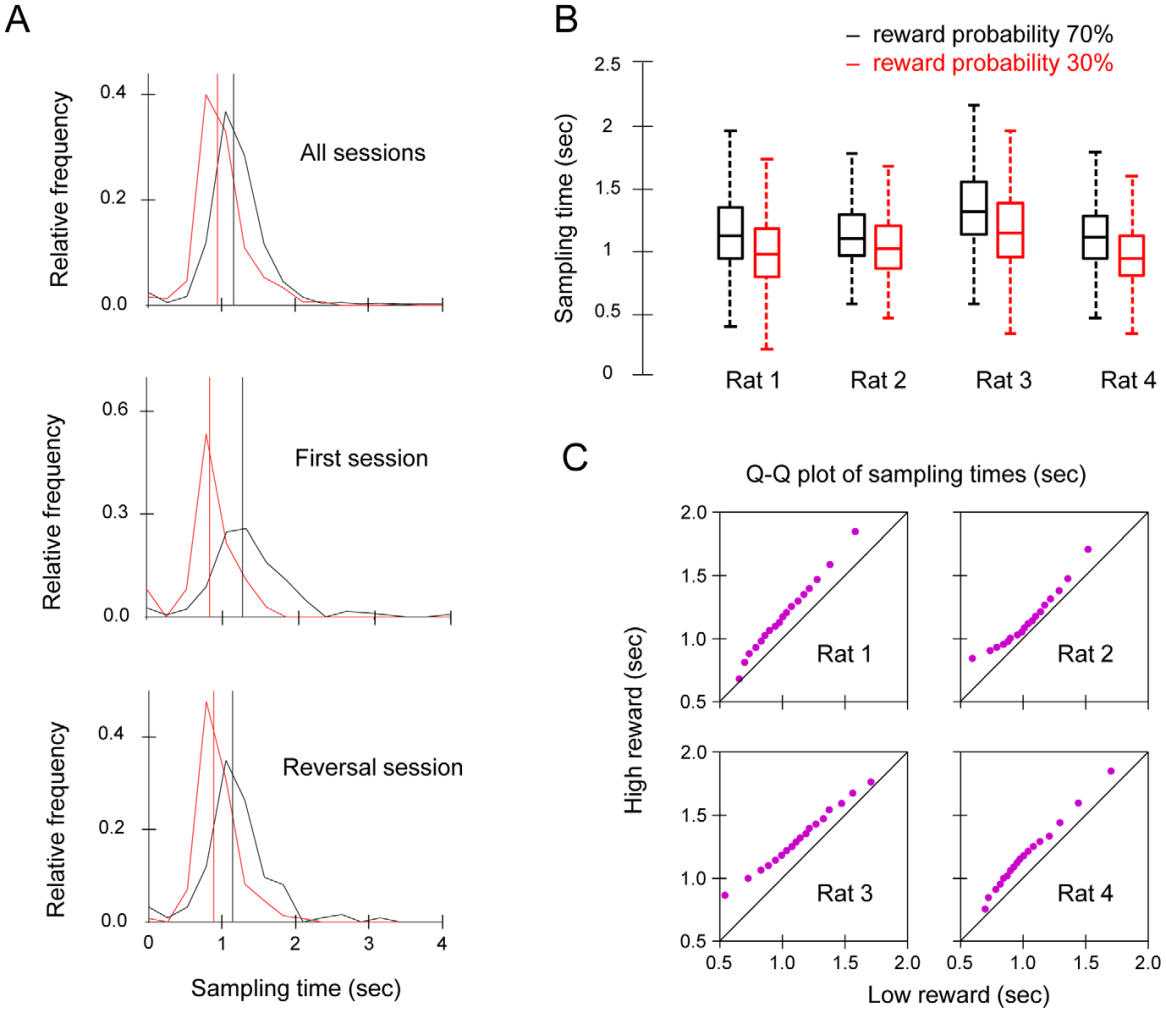
The distribution of times spent at spouts on unrewarded trials. A. Time spent at spout is defined as the time duration between the beginning of the first lick to the end of the last lick on each trial. Black vertical lines represent median times spent at high reward spout. Red vertical lines represent median times spent at low reward spout. Distributions are normalised for total number of trials. Top panel: Data from all sessions. Middle panel: Data from the first acquisition session only. Bottom Panel: Data from the first reversal session only. B. Box plot of data from all sessions, plotted per rat. The whiskers indicate the extent of the data (the minimum and maximum after excluding outliers), box and middle line indicate lower and upper quartiles and median, respectively. C. Quantile-quantile plots (Q-Q plots) per individual rat. Corresponding quantiles for high and low rewards spouts are calculated at 5% steps and plotted against one another. All points lie above the diagonal line, indicating that rats wait longer at the high than the low reward spout. However, there is no systematic deviation from linearity, suggesting no other systematic difference between the distribution of waiting times.

The key behavioural manipulation in the current experiment was the introduction of a variable time between the behavioural choice (i.e. first contact at one of the two spouts) and the disclosure of the outcome (i.e. presence or absence of reward). This allowed us to measure the time spent at the spout as a behavioural indication of the confidence in the outcome. For this analysis we exclusively examined the unrewarded trials where the experience of the outcome itself was identical. Figure 3A illustrates the distribution of times spent at the two spouts for the unrewarded trials across all sessions (top panel), the first acquisition session (middle panel), and the first session after reversal (lower panel). In every case, there was a lateral shift in the distribution of times spent at the high reward probability spout as also illustrated for individual rats in box and whisker plots of Figure 3B. This indicates a systematic rise in the average time spent at the high reward probability spout, with a mean increase of 173msec, across all rats and sessions. Across both reward probabilities, the time spent at the spout extended significantly beyond the expected reward onset window (i.e. maximum of 600 msec post-first-contact). In spite of this, the median value of each distribution (vertical lines in Figure 3A) were significantly different across sessions and rats (Wilcoxon signed-rank test p<0.001).

To further quantify the overlap between the sampling time distributions, we employed a quantile analysis (see Methods). Figure 3C illustrates the quantile-quantile plots (Q-Q plots) for each individual rat, for trials pooled across all sessions. The profiles indicate a consistent pattern of sampling whereby rats spent longer times on the high reward probability spout (i.e. each specific quantile is reached at a later time for the high probability spout).

**Figure 4:**
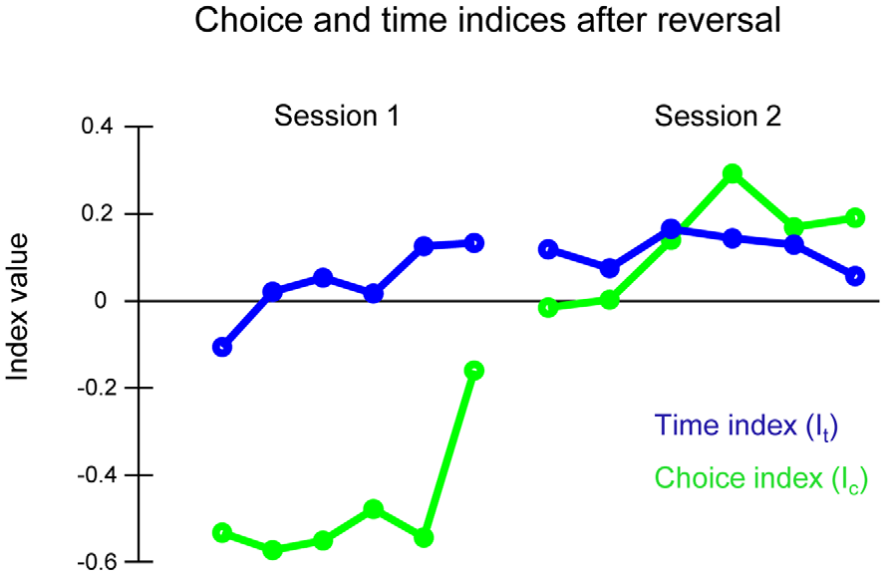
Comparison of the choice and time indices. A. The choice index (I_c_ – green lines) and time index (I_t_ – blue lines) in the first two sessions after reversal. The data points represent 50 successive and non-overlapping trials in a session. Data are averaged across all four rats. The time index passes 0 before the choice index, indicating that time spent at the high reward spout exceeds time spent at the low reward spout, long before the high reward spout is chosen more frequently.

Thus far the key finding, confirmed across multiple measures, was that rats adapted both their choice ratios and the time spent at spouts dynamically across trials. We next asked whether the two behavioural measures were correlated across trials and whether one of the two systematically preceded the other. In order to do this, we defined two behavioural indices, I_c_ and I_t_, to track the local choice ratios as well as the relative sampling times respectively. I_t_ captures the normalised difference in sampling time between the two spouts while I_c_ captures the relative choice employing a similar formula for direct comparison (see Methods). Figure 4 shows the choice and sampling time indices during the first two sessions of reversal when they both represent the fastest rate of change and their comparison is thus most informative. At the beginning of the first session, both choice ratios and sampling times reflect the acquisition contingencies – the rats select the newly low reward side on a bigger proportion of trials (average I_c_ is negative) and they spend longer times on the low reward spout (average I_t_ is negative). Interestingly, during session 1, the time ratios cross zero indicating the rats have begun to spend more time on the high reward spout. Note that the I_t_ is calculated based on the unrewarded trials only. For all successive sessions, the time index remained positive. In contrast, the choice index remained negative throughout the first session and became positive in the middle segment of the second session. These data indicate that differential sampling or waiting time occurs in advance of a change in the choice between the two locations. Across all sessions, the choice and time indices showed a significant level of correlation (Pearson Correlation Coefficient ranging from 0.57 to 0.67 across rats).

**Figure 5:**
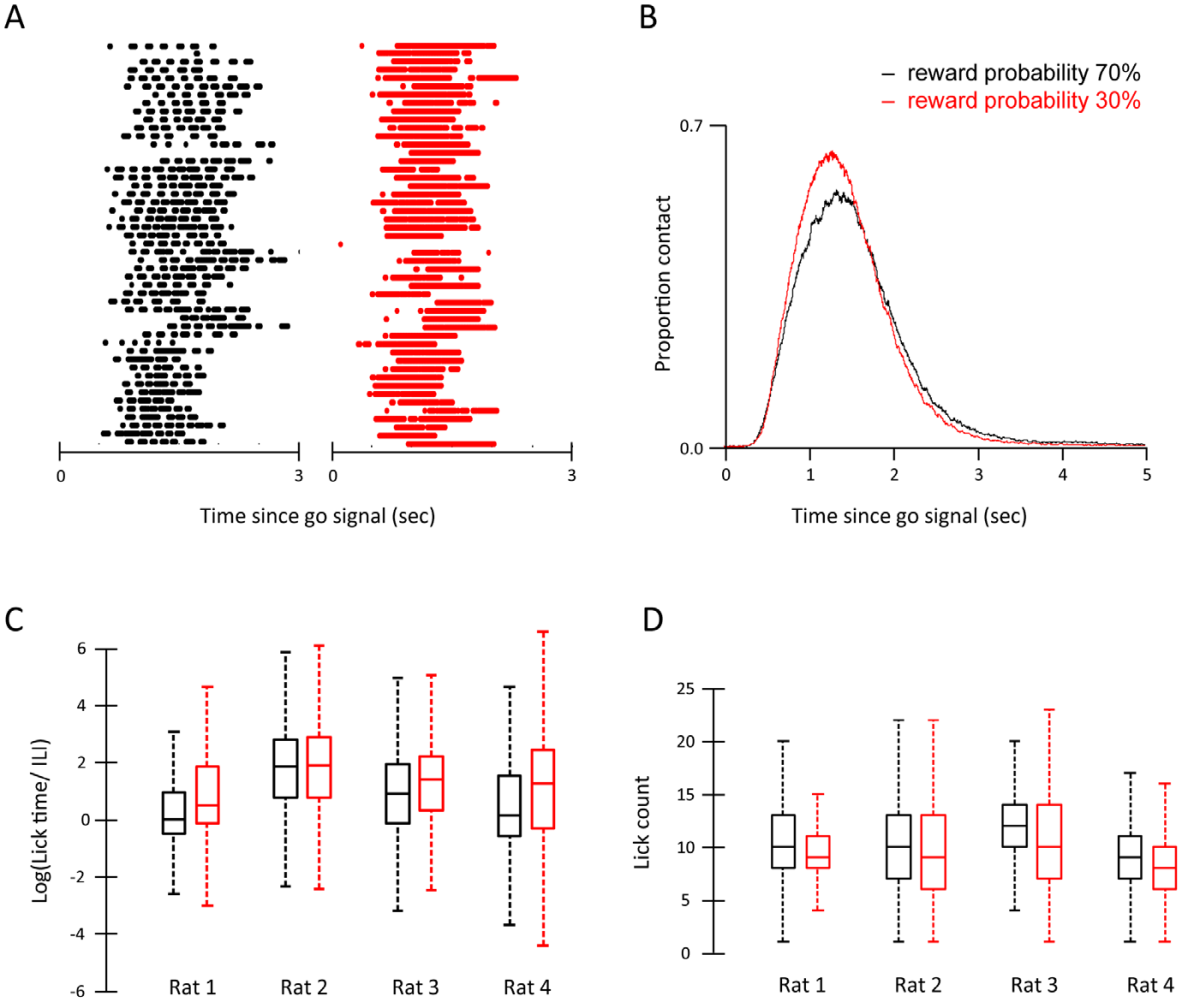
Licking profile and distribution. A. Sampled licking patterns are shown for rat 4. Every row indicates an individual trial. Licks were recorded at 1 msec precision. B. Average contact profiles for unrewarded trials, separated into high probability (black) and low probability (red) spouts. C. Box plots of log of lick density (natural logarithm); the ratio of individual lick duration to inter-lick interval, per rat, at the high probability (black) and low probability (red) spouts. Zero values indicate equal length, negative values indicate longer inter-lick interval durations and positive values indicate longer lick durations. D. Box plots of lick count per rat, at the high probability (black) and low probability (red) spouts. Box plots in C and D follow the convention described in Figure 3.

The key finding was that sampling or waiting times on unrewarded trials robustly indicated the relative reward probabilities across days, and that this differential pattern developed before any changes in the choice between the two locations. In light of this observation, we examined other aspects of the licking profile to search for further behavioural correlates of outcome probability. Figure 5A shows an example raster plot of contact profiles for the high probability trials in black and for the low probability trials in red, aligned by the go signal. Inspection of the contact profiles confirmed the previous finding of differential sampling duration for high versus low probability sides. Moreover, the microstructure of contacts revealed further differences: for the high reward condition (i) individual contacts were shorter and (ii) the interval between contacts was longer. The contact time histogram averaged across all rats (Figure 5B) captures the key findings. The contact profile at the low reward probability side was initially higher (red line) indicating denser contact patterns. At around 2000 msec after the go-signal, the profiles cross over with the high reward probability side showing more contacts, indicating longer sampling times at that spout. Across rats, individual contacts were significantly longer for the low reward spout (p value<0.01) with shorter inter-lick intervals (p value <0.01). To visualise the lick structure, Figure 5C gives the lick density, the log of the ratio of individual lick duration to inter-lick interval. In accordance with lick duration, there is a difference in lick patterns between high and low reward probability spouts; rats' licking was denser at the low reward spouts, consistent with the lick pattern statistics provided earlier. This difference was not significant for rat 2 where the ratio appears to have reached a ceiling (with lick durations on average 6.38 times higher than inter-lick interval for the high reward spout and 6.59 times longer than inter-lick interval for the low reward spout). Finally we examined the number of licks generated on each trial. Figure 5D illustrates the number of licks produced on each trial separately for the high and low reward probability spouts. In agreement with the sampling duration data, the average lick count was higher for the high reward probability spout for each of the four rats.

## Discussion

### A behavioural correlate of reward expectation

We used a discrete trial choice paradigm, where two options gave rewards at two distinct contingencies of 70% and 30%. Rats started by selecting the two options equally and eventually converged on nearly exclusive choice of the high probability option. After performing at a stable level of choice, the contingencies were reversed. All rats switched their choices back to the optimum behaviour (i.e. nearly exclusive choice of the new high probability reward) during 6 to 8 sessions. During every session, an analysis of the time spent at the spout revealed a systematic pattern whereby the rats spent more time at the high reward probability spout. This was the case when we exclusively examined the unrewarded trials, where the experience of the outcome itself was identical. Critically, the differential sampling times preceded the differential choice ratios. This was most evident in the examination of choice and time indices during the first session after reversal. This measure could thus be a predictor of the changes in choice and a potential correlate of a neuronal mechanism mediating the switch between the two options. Besides the total time duration spent at either spout, the profile of licking showed systematic differences across the high and low probability reward spouts. There were more licks for the high reward spout but individual licks were denser for the low reward spout.

One interpretation for the denser lick patterns at the low reward spout can be based on a property of ratio schedules. Such schedules make a reward contingent on the emission of either a fixed or variable number of responses. These schedules thus arrange a positive relation between the rate of responding and the rate of reward. Here, the contingencies were such that lick density also determined (by however small an amount) how quickly the outcome was procured (the outcome was presented at the first lick after the designated delay) and, hence, how quickly the rat could detect the absence of the outcome and leave the spout to initiate the next trial. The denser lick patterns exhibited by the rats when they selected the spout with the lower reward probability may have been reinforced more strongly compared to the high reward as they provided quicker feedback about the non-occurrence of the reward which occurred more frequently (70% non reward versus 30% non reward). This would thereby allow the rat to rapidly abandon the low probability spout and initiate the next trial.

### Matching versus maximising – statistical optimality and ecological benefit

Typically, when given a choice among possible options, animals exhibit two distinct modes of behaviour described as *matching* or *maximising* [6], [12], [13], [14]. In certain situations, animals distribute their choices proportional to the relative outcomes, a behaviour known as matching [8], [15], [16], [17]. Alternatively, animals exclusively choose one of the options – that with the higher probability of outcome – thereby increasing the relative occurrence of the outcome. This behaviour is termed maximising and is the statistically optimal behaviour – it garners the greatest outcome frequency. Both species [18], [19], [20] and experimental conditions seem to play a role in determining whether an animal employs matching or maximising [16]. Regardless of the evolutionary underpinning and relative optimality of these tactics, both require that animals represent the uncertainty and the probable outcome associated with a choice.

In settings where outcome probabilities are fixed and independent from sample to sample, maximising earns the highest reward. However, it has been argued [21] that if the contingencies of outcome change over time then sampling options with previously lower reward frequency is important. Moreover, if the choices from trial to trial are not independent (e.g. if the reward assigned to one option remains available until it is sampled) then the optimum behaviour approximates matching [22].

### Suboptimal sampling duration

On average, only 9.6% of trials were concluded at or before 600 msec (the latest time at which reward could become available) with the time spent at the spout being 700–1100msec (interquartile range). The time spent at the spout was therefore suboptimal and did not reflect learning of the precise onset of the reward across trials. To further investigate this pattern of behaviour, we studied the times spent at the spout during the rewarded trials. The average distribution of times spent at the spout for the rewarded trials was 2200–2900 msec (interquartile range) with no systematic differences across the two sides. Median time spent at the spout for rewarded trials was 2537 msec. The longer duration is due to the running of the sucrose pump during which the rat experiences the reward. When the conclusion time of the unrewarded trials were compared with the median time spent on the rewarded trials, 99% of the unrewarded trials were abandoned by that time. It is therefore possible that the moment by moment experience of reward during the rewarded trials, and not the reward onset drives the suboptimal sampling durations for unrewarded trials.

### Quantifying certainty of sensory decisions

In the current paradigm we have demonstrated that higher probabilities of reward cause the animals to sample the reward spout for longer durations indicating higher expectation of reward and confidence of an outcome. This phenomenon can be exploited in paradigms where uncertainty is not only governed by introducing an explicit probabilistic reward but affected by the uncertainty innate in sensory systems. For instance, in a tactile task involving rat whiskers [23] we found that the animals' discrimination performance at various vibrotactile intensities could be explained in terms of the response function of cortical neurons. Future experiments could apply the spout sampling measurement employed here in such sensory discrimination tasks in order to investigate whether time spent at the reward spout similarly reflects confidence in the neuronal representation of a sensory stimulus. Such an approach could be useful when other potential measures of confidence such as reaction times to sensory stimuli do not vary with task difficulty; for instance in the case of scent discrimination in rats [24]. Other attempts to quantify confidence in sensory discrimination tasks have involved introducing alternative discrete choices. Kepecs and colleagues employed a delayed reward version of an olfactory discrimination task [25]. Rats were given a short period of time after making a choice to either continue a trial or to abandon that trial and restart the subsequent one. Interestingly, the probability of reinitiating the next trial changed systematically as a function of performance reflecting the level of confidence in sensory integration. Similarly, a previous study trained Macaques in a visual task involving detecting the direction of moving random dots [26] and successfully quantified decision confidence by giving the animal a choice to opt out of the task for a small but certain reward. In both of these paradigms, the animal was trained to make an explicit alternative choice (i.e. either abandoning a trial within a fixed time window in the rat experiment or selecting a third option in the monkey experiment) when the confidence in the outcome was low. Our findings indicate that sampling duration at the choice spout, as well as the number and profile of licking could provide a direct measure of confidence in a simple two-alternative choice paradigm.

## Methods

### Ethics Statement

The experiments were conducted in accordance with the Australian and the international guidelines for the treatment of animals and were approved by the Animal Care and Ethics Committee at the University of New South Wales (ACEC number 10/47B).

### Subjects, behavioural apparatus and procedure

Subjects were four adult male 250–400 g Wistar rats. Rats were maintained on a 12:12 hour light-dark cycle (with lights on at 7 am) in a climate-controlled colony room. Rats were maintained on a mild food/water deprivation (12 g of rat chow, 3 hours of ad lib access to water each day) and were rewarded with a 5% sucrose solution during the experiment.

The experiment was performed in a Plexiglas chamber (30 cm in length×20 cm in width×50 cm in height) with a flooring of metal bars spaced at 1 cm. An aperture (40 mm×40 mm) was located in the front wall of the chamber. Nose-pokes into the aperture were detected by an infra-red optical sensor. Two light emitting diodes (LEDs) at the back wall of the nose-poke chamber were lit after a variable delay (100–600 ms uniform distribution) to indicate the go-signal. The reward was delivered through two drinking spouts located at either side of the aperture in the front wall (Figure 1). The behaviour of the rat (nose-poke or the response at either reward spout) was continuously registered into a data acquisition card (National Instruments, Inc., Austin, TX) using a custom-built circuit that measured contact at the spouts through closure of an electrical circuit or nose-poke through an optical sensor. A MATLAB script controlled the presentation of the go-signal, registered the behaviour of the rats along with the corresponding time stamp of each behavioural action, and controlled the delivery of rewards through two separate water pumps. The behaviour was additionally monitored during the experiment using an infrared camera positioned on the front wall of the aperture.

Figure 1 illustrates the basic experimental design and sequence of events in the task. The go-signal started with a variable delay after the nose-poke initiation, provided that the rat maintained the nose-poke throughout this delay. The rat then responded by choosing one of the two reward spouts which provided either 0.06 ml of sucrose water or nothing according to predetermined probabilities (0.7 or 0.3). The delivery of reward is independent from one trial to the next. The reward probabilities at the two spouts were statistically independent, such that sucrose could be available at both spouts (at a probability of 0.7*0.3 equal to 0.21) or at neither spout (at a probability of 0.3*0.7 equal to 0.21). The first lick at either drinking spout was considered as the behavioural choice and its time instance was recorded as the response time. If available on a spout, the reward was allocated at a delay from first spout contact (uniform distribution from 100 to 600 msec) and was delivered at the first lick after the designated time. The timing of the reward was independent of the predetermined probability.

After the familiarisation to the set up and the initial shaping of the behaviour, an acquisition period was conducted over 5 days. During the acquisition the contingencies were fixed for each rat (70% rewarded on one side and 30% on the other) and were fully counterbalanced across rats. Rats performed an average of 275 trials (218.5–329.5 inter-quartile range) during each session.

In the second phase (the reversal), the reward probabilities were reversed for each rat (a spout that was rewarded at a probability of 0.7 during acquisition, had a probability of 0.3 after reversal and vice versa for the other spout).

We defined two behavioural indices, the time index (I_t_) and the choice index (I_c_) to track the relative sampling times and the local choice ratios respectively. 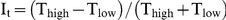



I_t_ reflects the normalised difference in sampling time (i.e. time duration from the beginning of the first contact to the end of the last contact) between the high and the low reward spouts. T_high_ is the median sampling duration at the high reward spout and T_low_ is the median sampling duration at the low reward spout during the window of interest after excluding the rewarded trials. A 50 trial window is chosen for the analysis reported in Figure 4. Similar results were found for a range of window sizes (20, 30, and 40; data not shown). 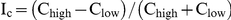



Similarly, I_c_ reflects the normalised choice difference between the two spouts across the same window of trials. C_high_ is number of times the high reward spout was chosen and C_low_ is number of times low reward spout was chosen during the window of interest. As before, the first lick at either drinking spout was considered as the behavioural choice.

### Quantile-Quantile analysis

To quantify the overlap between the sampling time distributions (Figure 3A), we found the quantile points of each distribution at 5% steps. Corresponding quantile values for each distribution were then plotted against each other to generate a Q-Q plot [27]. When the two distributions being compared are identical, the dots on a Q-Q plot fall on the diagonal line. If one distribution is simply shifted laterally compared to the other, then the dots will fall on a parallel line above or below the diagonal. Any change in the angle of the line relative to the diagonal or deviation from linearity would indicate other differences between the two distributions (differences in distribution width, skewness, or shape).

## References

[1] Krebs JR (1977). Optimal foraging: Theory and experiment.. Nature.

[2] Healy SD, Hurly TA (2003). Cognitive ecology: Foraging in hummingbirds as a model system.. Advances in the Study of Behavior.

[3] Pyke GH (1984). Optimal foraging theory: a critical review.. Annual Review of Ecology and Systematics.

[4] Pyke GH, Pulliam HR, Charnov EL (1977). Optimal foraging: a selective review of theory and tests.. The Quarterly Review of Biology.

[5] Hayden BY, Pearson JM, Platt ML (2011). Neuronal basis of sequential foraging decisions in a patchy environment.. nature neuroscience.

[6] Mazur JE (2010). Distributed versus exclusive preference in discrete-trial choice.. Journal of Experimental Psychology: Animal Behavior Processes.

[7] Kacelnik A, Bateson M (1996). Risky theories—the effects of variance on foraging decisions.. American Zoologist.

[8] Sugrue LP, Corrado GS, Newsome WT (2004). Matching behavior and the representation of value in the parietal cortex.. Science.

[9] Krebs JR, Davies NB (1997). Behavioral ecology: Wiley-Blackwell.

[10] Herrnstein RJ, Rachlin H, Laibson DI (1997). The matching law: papers in psychology and economics..

[11] Sakai Y, Fukai T (2008). The actor-critic learning is behind the matching law: Matching versus optimal behaviors.. Neural Computation.

[12] Baum WM (1974). On two types of deviation from the matching law: Bias and undermatching.. Journal of the Experimental analysis of Behavior.

[13] Baum WM (1979). Matching, undermatching, and overmatching in studies of choice.. Journal of the Experimental analysis of Behavior.

[14] Davison M, McCarthy D (1988). The matching law: A research review: Lawrence Erlbaum Associates, Inc.

[15] Foster TA, Hackenberg TD (2004). Unit price and choice in a token-reinforcement context.. Journal of the Experimental analysis of Behavior.

[16] Graf V, Bullock D, Bitterman M (1964). Further experiments on probability-matching in the pigeon.. Journal of the Experimental analysis of Behavior.

[17] Woolverton WL, Rowlett JK (1998). Choice maintained by cocaine or food in monkeys: effects of varying probability of reinforcement.. Psychopharmacology.

[18] Bitterman M, Wodinsky J, Candland DK (1958). Some comparative psychology.. The American Journal of Psychology.

[19] Meyer DR (1960). The effects of differential probabilities of reinforcement on discrimination learning by monkeys.. Journal of Comparative and Physiological Psychology.

[20] Bullock DH, Bitterman M (1962). Probability-matching in the pigeon.. The American Journal of Psychology.

[21] Lea S (1979). Foraging and reinforcement schedules in the pigeon: Optimal and non-optimal aspects of choice.. Animal Behaviour.

[22] Shimp CP (1969). Optimal behavior in free-operant experiments.. Psychological Review.

[23] Adibi M, Arabzadeh E (2011). A comparison of neuronal and behavioral detection and discrimination performances in rat whisker system.. Journal of Neurophysiology.

[24] Uchida N, Kepecs A, Mainen ZF (2006). Seeing at a glance, smelling in a whiff: rapid forms of perceptual decision making.. Nature Reviews Neuroscience.

[25] Kepecs A, Uchida N, Zariwala HA, Mainen ZF (2008). Neural correlates, computation and behavioural impact of decision confidence.. Nature.

[26] Kiani R, Shadlen MN (2009). Representation of confidence associated with a decision by neurons in the parietal cortex.. Science.

[27] Wilk MB, Gnanades R (1968). Probability Plotting Methods for Analysis of Data.. Biometrika.

